# Voltage-Sensitive K^+^ Channels Inhibit Parasympathetic Ganglion Transmission and Vagal Control of Heart Rate in Hypertensive Rats

**DOI:** 10.3389/fneur.2015.00260

**Published:** 2015-12-08

**Authors:** Torill Berg

**Affiliations:** ^1^Division of Physiology, Department of Molecular Medicine, Institute for Basic Medical Sciences, University of Oslo, Oslo, Norway

**Keywords:** hypertension, parasympathetic ganglia, sympathetic ganglia, norepinephrine release, acetylcholine release, heart rate, voltage-sensitive K^+^-channels, 3,4-diaminopyridine

## Abstract

Parasympathetic withdrawal plays an important role in the autonomic dysfunctions in hypertension. Since hyperpolarizing, voltage-sensitive K^+^ channels (*K*_V_) hamper transmitter release, elevated *K*_V_-activity may explain the disturbed vagal control of heart rate (HR) in hypertension. Here, the *K*_V_ inhibitor 3,4-diaminopyridine was used to demonstrate the impact of *K*_V_ on autonomic HR control. Cardiac output and HR were recorded by a flow probe on the ascending aorta in anesthetized, normotensive (WKY), and spontaneously hypertensive rats (SHR), and blood pressure by a femoral artery catheter. 3,4-diaminopyridine induced an initial bradycardia, which was greater in SHR than in WKY, followed by sustained tachycardia in both strains. The initial bradycardia was eliminated by acetylcholine synthesis inhibitor (hemicholinium-3) and nicotinic receptor antagonist/ganglion blocker (hexamethonium), and reversed to tachycardia by muscarinic receptor (mAchR) antagonist (atropine). The latter was abolished by sympatho-inhibition (reserpine). Reserpine also eliminated the late, 3,4-diaminopyridine-induced tachycardia in WKY, but induced a sustained atropine-sensitive bradycardia in SHR. Inhibition of the parasympathetic component with hemicholinium-3, hexamethonium, or atropine enhanced the late tachycardia in SHR, whereas hexamethonium reduced the tachycardia in WKY. In conclusion, 3,4-diaminopyridine-induced acetylcholine release, and thus enhanced parasympathetic ganglion transmission, with subsequent mAchR activation and bradycardia. 3,4-diaminopyridine also activated tachycardia, initially by enhancing sympathetic ganglion transmission, subsequently by activation of norepinephrine release from sympathetic nerve terminals. The 3,4-diaminopyridine-induced parasympathetic activation was stronger and more sustained in SHR, demonstrating an enhanced inhibitory control of *K*_V_ on parasympathetic ganglion transmission. This enhanced *K*_V_ activity may explain the dysfunctional vagal HR control in SHR.

## Introduction

It is generally accepted that hypertension is associated with sympathetic hyperactivity and parasympathetic hypoactivity (1–3), and a high resting heart rate (HR) is the most reliable predictor of cardiovascular morbidity and hypertension in human (4, 5). Sympathetic control of HR is on a beat-to-beat basis controlled by the baroreflex, activated by a rise in blood pressure (BP). Information from the baroreceptors is mediated to the nucleus tractus solitarii, leading to downregulation of sympathetic output from the rostral ventrolateral medulla as well as to the stimulation of the nucleus ambiguous with subsequent activation of efferent vagal nerves to the heart. Thus, HR is controlled by both inhibitory parasympathetic vagal nerves and stimulatory sympathetic nerves. The elevated HR in hypertension may therefore result from an insufficient vagal inhibition of the sympathetic activity.

Autonomic dysregulation is also a characteristic feature of heart failure, manifested by increased sympathetic activity and reduced parasympathetic activity (6). Abnormalities in the vagal control of HR may be directly responsible for a poor outcome in myocardial infarction (7). In heart failure, there is evidence in animals and humans to indicate that the parasympathetic ganglia act as a bottleneck to efferent vagal traffic (8). It may therefore be hypothesized that parasympathetic ganglia are responsible for a dysfunctional vagal control of HR also in hypertension.

A major component of the parasympathetic control of HR involves inhibition of sympathetic activation, i.e., sympathetic activity acts as a substrate for vagal inhibition (9). Analysis of the sympathetic–parasympathetic interaction in the control of HR therefore requires both systems to be activated simultaneously. Dual control is not easily activated in the anesthetized rat but was achieved by 4-aminopyridine (4-AP) (10). 4-AP blocks voltage-sensitive K^+^ channels (*K*_V_) and therefore depolarizes neurons, and, through that, it opens voltage-sensitive Ca^2+^ channels. The resulting entry of Ca^2+^ activates neuronal transmitter release. Similar events stimulate Ca^2+^-induced contraction in vascular smooth muscle cells (VSMCs). 4-AP-injected IV in normotensive rats (WKY) therefore induced a transient rise in TPR. It also induced bradycardia due to release of acetylcholine (Ach) from parasympathetic nerves in WKY but not in spontaneously hypertensive rats (SHR). The initial response was in both strains followed by a sustained tachycardia, which was abolished by reserpine and was therefore due to norepinephrine (NE) release from peripheral sympathetic nerves (10). The nicotinic receptor (nAchR) antagonist hexamethonium eliminated the initial 4-AP-induced bradycardia in WKY and reversed the bradycardia to tachycardia in SHR, suggesting that the initial parasympathetic component resulted from activation of parasympathetic ganglion transmission. However, hexamethonium did not alter the late tachycardia in either strain, although a minor, but prolonged atropine sensitive, parasympathetic component was revealed in SHR when the sympathetic tachycardia had been eliminated (10). The response to 4-AP was therefore largely dominated by activation of NE release in SHR, making it difficult to analyze the mechanisms involved in the parasympathetic control of HR and to evaluate strain-related differences in this component.

**Figure 1:**
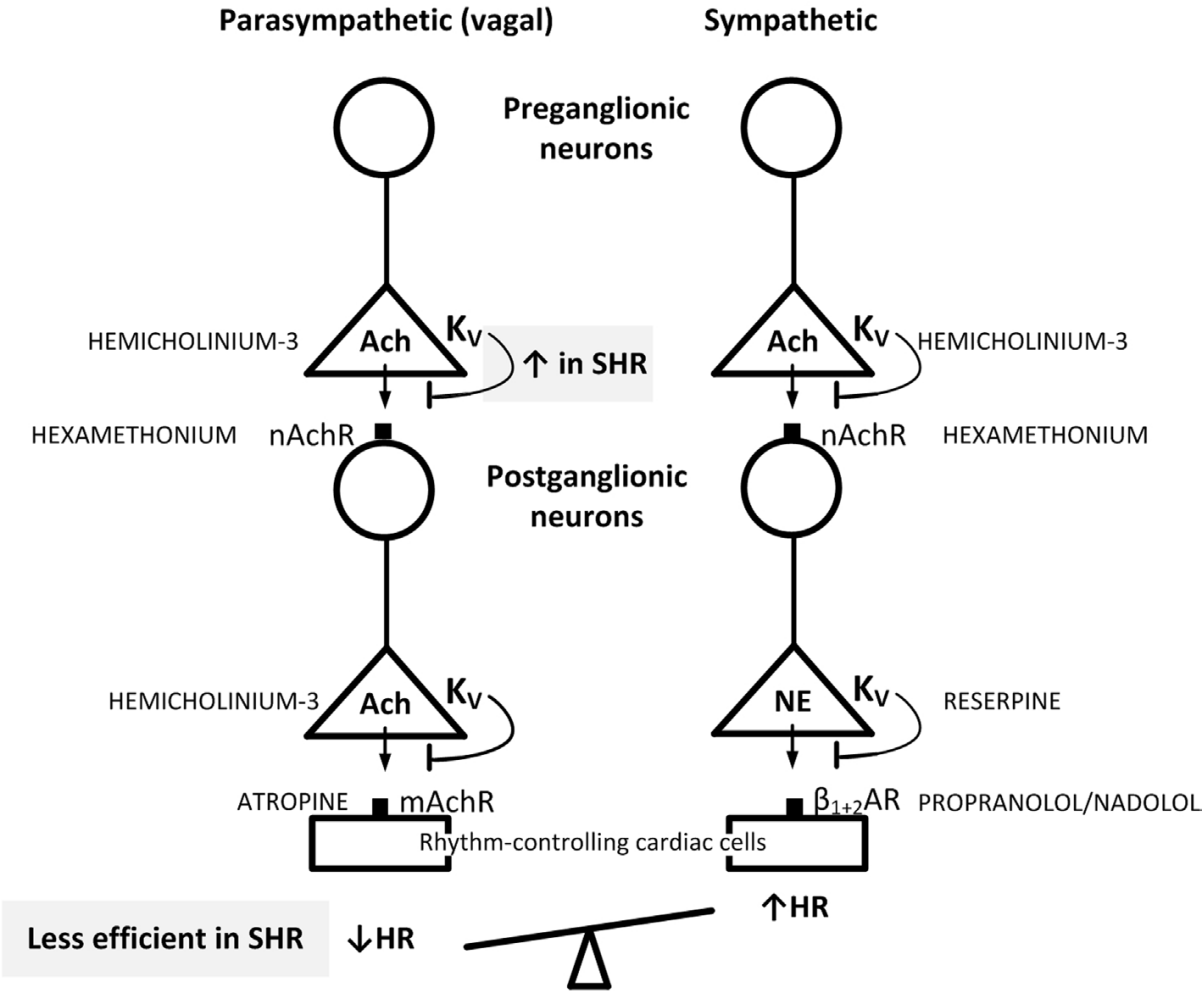
**Suggested localization of the inhibitory effect of 3,4-DAP on *K*_V_ in pre- and postganglionic parasympathetic and sympathetic neurons involved in the regulation of HR**. By inhibiting *K*_V_, 3,4-DAP will induce Ach release from preganglionic parasympathetic and sympathetic nerve endings as well as in postganglionic parasympathetic nerve terminals, and also induce norepinephrine (NE) release from postganglionic sympathetic nerve endings. The inhibitors used to localize the effect of 3,4-DAP, i.e., the Ach synthesis inhibitor hemicholinium-3, the mAchR and nAchR blockers atropine and hexamethonium, respectively, reserpine which depletes sympathetic nerve endings of NE, and the βAR antagonist nadolol, which block sympathetic control of HR, are indicated in capital letters next to site of action. When effect of one drug involved more than one step in the response chain or involved both the parasympathetic and sympathetic chains, drugs were combined to identify the site of 3,4-DAP action. Through the effect on HR, the results showed that Ach release from preganglionic vagal nerves was blunted by augmented 3,4-DAP-sensitive hyperpolarizing *K*_V_ activity in SHR (indicated in upper gray box), thus preventing activation of the postganglionic neuron and vagal inhibition of HR (indicated in lower gray box). Presynaptic *K*_V_ in the parasympathetic ganglia therefore functioned as a bottleneck in vagal transmission in SHR. These ganglia are likely to be located in superficial fat tissue close to the sinoatrial node. Blunted arrows indicate the inhibitory effect of presynaptic *K*_V_ on transmitter release.

4-Aminopyridine easily enters the central nervous system (CNS) (11, 12), where it may increase central sympathetic output by activating release of Ach (11, 13). This activation may influence the peripheral effects of 4-AP on parasympathetic and sympathetic ganglion transmission and neuronal release. In a previous study (14), another *K*_V_ inhibitor, i.e., 3,4-diaminopyridine (3,4-DAP), which does not cross the blood–brain barrier (12, 15), was found to be more efficient than 4-AP in producing an initial bradycardia in SHR. It was also far more efficient in activating salivation (15), suggesting that it may be a better parasympathetic activator than 4-AP. In the present study, 3,4-DAP was therefore used to explore if the impact of *K*_V_ on autonomic HR control in SHR differed from that in WKY. By use of pharmacological intervention prior to 3,4-DAP as out-lined in Figure 1, two hypothesis were tested: (1) if the parasympathetic component involved in the HR response to 3,4-DAP resulted from activation of parasympathetic ganglion transmission, with release of Ach from the preganglionic neuron and activation of postsynaptic nAchR, leading to stimulation of muscarinic receptors (mAchRs) in rhythm-controlling effector cells, such as the sinoatrial node. (2) If augmented hyperpolarizing *K*_V_ currents hampered the release of Ach in parasympathetic ganglia in SHR, thus precipitating the parasympathetic withdrawal and altered autonomic control of HR associated with hypertension.

## Materials and Methods

### Surgical Procedure

All experiments were approved by The Norwegian Animal Research Authority (NARA), project license no FOTS 2914, and were conducted in accordance with the European Directive 2010/63/EU. The experiments included 12–14 weeks old, male SHR (Okamoto, SHR/NHsd strain, *n* = 94, 299 ± 3 g body weight) and WKY (Wistar Kyoto, *n* = 72, 298 ± 4 g body weight) on conventional rat chow diet (0.7% NaCl). The rats were anesthetized with pentobarbital (65–75 mg/kg IP), and a satisfactory level of surgical anesthesia was verified by non-responsiveness to pinching between the toes. The rats were tracheotomized, and a heparinized catheter was inserted into the femoral artery for the measurement of systolic (SBP) and diastolic (DBP) BP and HR. After the starting BP and HR had been recorded, the rats were connected to a positive-pressure respirator and ventilated with air throughout the experiment. The thoracic cavity was entered through the third intercostal space, and a 2SB perivascular flow probe, connected to a T206 Ultrasonic Transit-Time Flowmeter (Transonic Systems Inc., Ithaca, NY, USA), was placed on the ascending aorta to measure cardiac output (CO, i.e., without cardiac flow) and from now on also HR. The thoracic cavity was subsequently closed with a suture. Body temperature was maintained at 37–38°C by external heating, guided by a thermo sensor carefully inserted inguinally into the abdominal cavity. When surgery was completed, the arterial catheter was flushed with 0.15 ml buffered saline (PBS; 0.01M Na-phosphate, pH 7.4, 0.14M NaCl) containing 500 IU/ml heparin. Drugs were dissolved in PBS and administered as bolus injections through a catheter in the femoral vein (0.6–1.3 ml/kg), unless otherwise indicated. A stabilizing period of 10 min was allowed before the first experimental drug was injected.

### Experimental Protocols

#### Protocol 1: The Cardiovascular Response to 3,4-DAP

Control rats were pre-treated with a sham injection containing vehicle (PBS) and 10 min later injected with the *K*_V_ blocker 3,4-DAP (34.5 μmol/kg) to stimulate dual activation of the autonomic nervous system. The cardiovascular response was then monitored for 25 min. To study the role of Ach release from preganglionic and postganglionic nerve endings as outlined in Figure 1, the PBS-sham injection was substituted with hemicholinium-3 (17.4 μmol/kg), which blocks the rate limiting step in Ach synthesis, i.e., re-uptake of choline through the high-affinity choline transporter. Hemicholinium-3 does not penetrate the blood–brain barrier (16). Involvement of parasympathetic/sympathetic ganglion transmission was studied by pre-treatment with the peripherally restricted (17), non-selective nAchR antagonist/ganglion blocker hexamethonium chloride (37 μmol/kg) (18). To evaluate activation of postganglionic, postsynaptic mAchR, the rats were pre-treated with atropine sulfate (6.9 μmol/kg, −20 min). As indicated in Figure 1, the impact of transmitter release from sympathetic nerve terminals was identified by pre-treatment with reserpine (8.2 μmol/kg IP, -48 and -24 h) (15), which depletes sympathetic nerve endings of NE. The effect of β-adrenergic blockade was studied by pre-treatment with nadolol (8.5 μmol/kg 10 min before 3,4-DAP) (19), a β_1+2_AR antagonist that does not penetrate the blood–brain barrier. Nadolol was administered alone in both strains and in SHR also 10 min after injection of atropine as above. In addition, SHR was pre-treated with quinidine (46.2 μmol/kg) (20), a peripherally restricted (21), class Ia antiarrhythmic drug, which also blocks K^+^ channels, such as the 4-AP sensitive *K*_V_1.5 (22, 23) and the muscarinic K_Ach_ (Kir3.1/Kir3.4) (24).

#### Protocol 2: The Cardiovascular Response to 4-AP Compared to 3,4-DAP

This protocol was similar to that in Protocol 1, but 3,4-DAP was substituted with an equimolar injection of 4-AP (34.5 μmol/kg) (10, 15). To demonstrate the role of Ach release in the response to 4-AP, the rats were pre-treated with hemicholinium-3 as above.

#### Protocol 3: The Cardiovascular Response to Nicotine

The postsynaptic nAchR in parasympathetic and sympathetic postganglionic neurons can be stimulated directly by nicotine (Figure 1). To investigate if the ability of these nAchR to respond differed in the two strains, WKY and SHR were injected with the agonist nicotine (1.8 μmol/kg), and the cardiovascular response was monitored for 15 min. To study if 3,4-DAP modified nAchR function in SHR, nicotine was injected 25 min after 3,4-DAP.

### 3,4-DAP- and 4-AP-Induced Salivary Flow and Glandular Kallikrein Secretion

Salivation does not occur in anesthetized rats but can be stimulated, here with 3,4-DAP or 4-AP, due to the activation of transmitter release from autonomic nerves. Nicotine does not activate salivation. Whole saliva was collected from the oral cavity with a pipette throughout the 4-AP- and 3,4-DAP-observation period. Saliva volume was recorded by weight. Saliva was stored at −20°C until assayed for S2266-kallikrein-like enzyme activity as an indication of sympathetic activation, since submandibular gland kallikrein in the rat is massively released upon α_1_-adrenergic stimulation (25). In short, saliva, diluted in PBS (100 μl total), together with 800 μl assay buffer (0.2 mol/l Tris/HCl buffer, pH 9.0) were incubated up to 5 min at 37°C with 2 mM S2266 substrate (26). The reaction was stopped with 100 μl of 50% (v/v) acetic acid, and absorption was measured at 405 nm.

### Measurement of Plasma Catecholamines

A total of 1.5 ml blood was collected at the end of the experiment by free flow from the femoral artery catheter into tubes containing 45 μl 0.2M glutathione and 0.2M EGTA (4°C). Plasma was stored at −80°C until catecholamine concentrations were determined using 400 μl plasma and the 5000 Reagent kit for HPLC analysis of Catecholamines in plasma from Chromsystems GmbH, Munich, Germany, as previously described (27).

### Drugs

Pentobarbital was obtained from The Norwegian National Hospital, Oslo, Norway; and S2266 from Kabi Diagnostica (Stockholm, Sweden). The remaining drugs were from Sigma Chemical Co., St. Louis, MO, USA.

### Statistical Analyses

The results are presented as mean values ± SEM. The cardiovascular data recorded throughout the experiments were averaged every minute, except during the initial response to 3,4-DAP, 4-AP, or nicotine, where data were averaged every seventh heartbeat. The cardiovascular response to pre-treatment, baselines prior to 3,4-DAP and 4-AP, salivary flow, and the salivary kallikrein and plasma catecholamine concentrations were evaluated overall by one-way ANOVA, including all groups within each strain in each protocol. When the presence of group differences was indicated, these were located by two-tailed, two-sample Student’s *t*-tests for parametric data, and by Kruskal–Wallis tests for non-parametric data. The HR response to 3,4-DAP/4-AP was recorded at the initial TPR peak response, i.e., at about 1–1.5 min, and during the late response, i.e., at 10, 15, 20, and 25 min. The response to nicotine was recorded at the HR nadir and the TPR peak response within the first minute and subsequently every minute throughout the observation period. Since TPR is determined by the vessel radius in the fourth power, changes in TPR were expressed in percent of before values. The 3,4-DAP/4-AP/nicotine-response curves were analyzed using repeated measures analyses of variance and covariance, first as overall tests including all groups within each strain and each protocol, and subsequently between groups or for each group separately. Significant responses (two-tailed, one-sample Student’s *t*-tests) and group differences (two-tailed two-sample Student’s *t*-test or Kruskal–Wallis tests) were subsequently located at specific times, i.e., during the initial response and at the end of the experiment. Testing proceeded only when the presence of significant responses, differences, and/or interactions was indicated. The *P*-value was for all tests and each step adjusted according to Bonferroni, except for salivary flow, the salivary kallikrein and plasma catecholamine concentrations, where *P* ≤ 0.05 was considered significant.

## Results

### Effect of Pre-Treatment on Cardiovascular Baselines

MBP and HR before the rats were connected to the ventilator were 67 ± 4 and 143 ± 10 mm Hg and 279 ± 12 and 376 ± 9 bpm in the WKY and SHR controls, respectively (*P* < 0.001 for strain-related differences). When connected to the ventilator and thoracotomized, BP remained not much different in WKY but was reduced in SHR because of a reduction in the venous return to the right heart due to the intrathoracic pressure. However, there was little change in HR. Still MBP remained higher in SHR than in WKY (Table 1). Also, baseline HR and TPR prior to 3,4-DAP were higher in the SHR controls than in the WKY controls (*P* = 0.007).

**Table 1:** **Cardiovascular baselines prior to 3,4-DAP, i.e., after pre-treatment, and the response to pre-treatment below in parenthesis**.

Pre-treatment		WKY			SHR		
		MBP (mm Hg)	HR (bpm)	TPR (mm Hg/ml/min)	MBP (mm Hg)	HR (bpm)	TPR (mm Hg/ml/min)
PBS	Vehicle	65 ± 5 (1 ± 1)	301 ± 15 (−7 ± 5)	2.10.1 (−0.1 ± 0.0)	84 ± 6* (−5 ± 4)	381 ± 12* (−8 ± 9)	5.2 ± 0.6* (−0.7 ± 0.2)
Hemicholinium-3	Ach synthesis inhibitor	58 ± 4 (−26 ± 4)^†^	303 ± 16 (−5 ± 8)	2.1 ± 0.1 (−0.5 ± 0.1)^†^	60 ± 7 (−21 ± 7)	354 ± 14 (−43 ± 9)^†^	3.1 ± 0.2^†^ (−0.9 ± 0.3)
Hexamethonium	nAchR antagonist	39 ± 5^†^ (−29 ± 6)^†^	257 ± 12 (−53 ± 14)^†^	1.7 ± 0.1 (−0.7 ± 0.2)^†^	53 ± 2^†^ (−33 ± 7)^†^	345 ± 19 (−67 ± 8)^†^	2.9 ± 0.2^†^ (−1.5 ± 0.2)^†^
Atropine	mAchR antagonist	68 ± 10 (−2 ± 3)	307 ± 15 (19 ± 12)	1.7 ± 0.2 (−0.3 ± 0.1)	76 ± 4 (5 ± 7)	374 ± 13 (−33 ± 6)	3.9 ± 0.3 (−0.1 ± 0.2)
Reserpine^a^ + PBS	Depletes norepinephrine	58 ± 3 (0 ± 0)	307 ± 4 (−14 ± 4)	2.0 ± 0.1 (0.0 ± 0.0)	62 ± 4 (1 ± 2)	309 ± 12^†^ (−23 ± 5)	4.1 ± 0.3 (0.4 ± 0.1)^†^
Reserpine + atropine		66 ± 4 (−7 ± 2)^†^	328 ± 9 (5 ± 12)	1.9 ± 0.1 (−0.3 ± 0.1)	73 ± 6 (1 ± 2)	278 ± 8^†^ (−19 ± 11)	4.3 ± 0.4 (−0.4 ± 0.3)
Nadolol	β_1+2_AR antagonist	61 ± 4 (2 ± 1)	312 ± 6 (−10 ± 2)	2.0 ± 0.2 (0.0 ± 0.1)	69 ± 5 (−8 ± 3)	343 ± 6 (−67 ± 25)^†^	3.9 ± 0.3 (−0.1 ± 0.2)
Atropine + nadolol			Not done		61 ± 9 (−9 ± 4)	325 ± 12 (−33 ± 6)	3.8 ± 0.2 (−0.0 ± 0.2)
Quinidine	Antiarrhythmic + *K*_V_/*K*_Ach_ inhibitor		Not done		67 ± 5 (−42 ± 9)^†^	313 ± 14 (−63 ± 13)^†^	2.5 ± 0.4^†^ (−1.2 ± 0.4)

^a^Since reserpine was administered prior to the experiment, the effect of reserpine is indicated by the differences in baselines. Comparisons were made between the WKY and SHR controls (*) and between the PBS control and experimental groups within each strain (^†^). Sixteen rats were included in the WKY and SHR controls, and 6–9 rats in the experimental groups.

*P ≤ 0.017.

*^†^*P* ≤ 0.006*.

Hemicholinium-3 reduced baseline MBP and TPR in WKY and HR in SHR (*P* ≤ 0.01). Hexamethonium reduced MBP, HR and TPR in both strains, whereas atropine had no effect. Reserpine and nadolol reduced HR in SHR. Quinidine reduced MBP and HR (*P* ≤ 0.007) (tested in SHR only).

### The HR Response to 3,4-DAP

**Figure 2:**
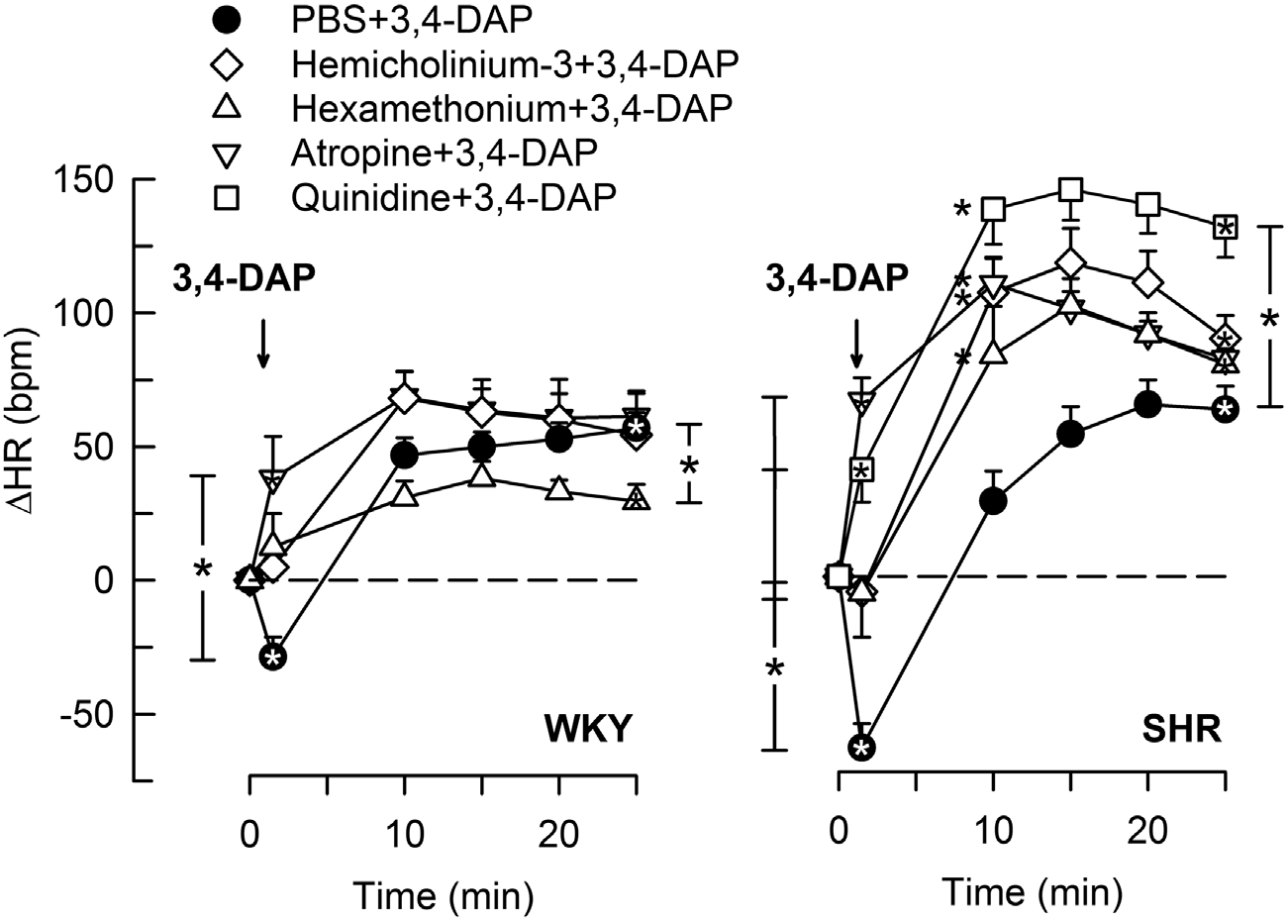
**The HR response to 3,4-DAP after pre-treatment with cholinergic inhibitors or quinidine**. Quinidine was given to SHR only. Significant responses (* within symbol) and group differences (* in brackets) at HR-nadir (after about 1 min, brackets left of curves) and at 25 min (right of curves) were located as indicated. *Left of symbol at 10 min in SHR indicates significant differences compared to the control group. *P < 0.0167 after curve analyses (please see Section “Materials and Methods” for details).

3,4-Diaminopyridine induced an immediate bradycardia (*P* ≤ 0.002), which was greater in SHR than in WKY (*P* = 0.005) (Figure 2). Subsequently, HR increased, and a sustained tachycardia was observed (*P* < 0.0001 at 25 min) with no significant strain-related difference after 25 min (Figure 2).

#### The Immediate HR Response to 3,4-DAP

**Figure 3:**
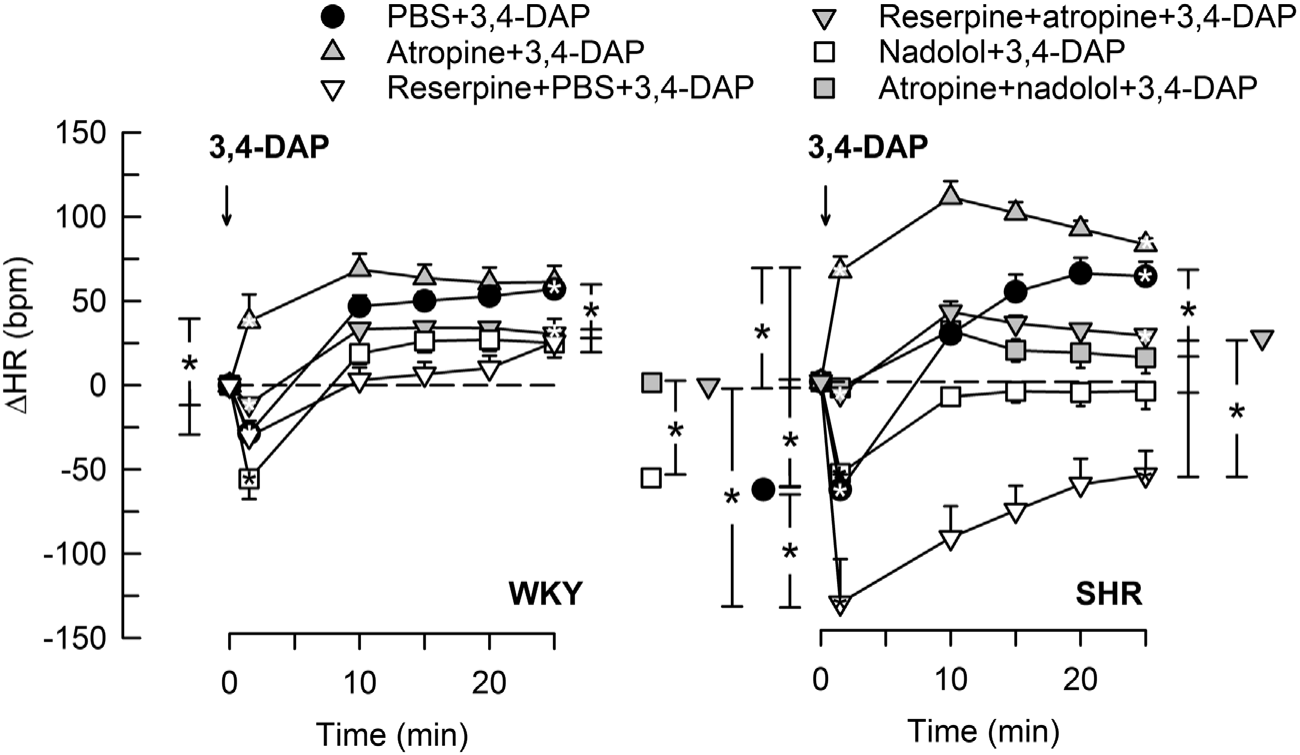
**The HR response to 3,4-DAP after pre-treatment with reserpine or nadolol, alone or combined with atropine**. Atropine + nadolol was given to SHR only. Significant responses (* within symbol) and group differences (* in brackets) at 1 (left of curves) and 25 min (right of curves) were located as indicated. Comparisons were made between the control and the experimental groups, and between the groups pre-treated with reserpine/atropine and reserpine + atropine, and between the nadolol- and atropine + nadolol-treated groups. *P < 0.025 after curve analyses.

Pre-treatment with the Ach synthesis inhibitor hemicholinium-3 or the nAchR antagonist hexamethonium eliminated the immediate HR response to 3,4-DAP in both strains (Figure 2). After the mAchR antagonist atropine, the initial 3,4-DAP induced bradycardia was reversed to tachycardia in both strains (*P* ≤ 0.01 one-sample Student’s *t*-test, *P* = 0.003 compared to the controls) (Figure 2). As outlined in Figure 1, these results demonstrated that 3,4-DAP-activated Ach release from the preganlionic neuron, thus activating the postsynaptic nAchR in parasympathetic postganglionic neuron, subsequently stimulating mAchR on rhythm-controlling cells. Elimination of the sympathetic component by pre-treatment with reserpine, enhanced the immediate, 3,4-DAP-induced bradycardia in SHR but had no effect on the bradycardia in WKY (Figure 3). When reserpine was combined with atropine, the bradycardia was eliminated in SHR (*P* ≤ 0.002 compared to the control and reserpine-only groups), showing that the bradycardia depended on parasympathetic activation. However, an initial tachycardia, as observed after atropine alone, was not observed (*P* = 0.001 compared to that after atropine alone). The same pattern was seen in WKY (Figure 3). This observation demonstrated that the initial tachycardia in atropine-treated rats was due to activation of sympathetic ganglion transmission and mediated through sympathetic nerves. The peripherally restricted β_1+2_AR antagonist nadolol had no significant effect on the initial 3,4-DAP-induced bradycardia in either strain (Figure 3). After pre-treatment with atropine + nadolol, 3,4-DAP produced no initial HR response (tested in SHR only), similar to that seen after reserpine + atropine in this strain. Pre-treatment with quinidine, similar to that after atropine, reversed the initial 3,4-DAP-induced bradycardia to tachycardia (*P* = 0.021) (tested in SHR only, Figure 2).

#### The Late HR Response to 3,4-DAP

When sympathetic nerve endings had been depleted of NE by reserpine (Figure 1), the 3,4-DAP-induced late tachycardia was abolished in WKY (*P* = NS) and reversed to a prominent bradycardia in SHR (*P* = 0.005 at 25 min, *P* < 0.001 compared to the controls) (Figure 3). These results showed that the late tachycardia depend on sympathetic nerve transmitter release in both strains. The reserpine-dependent bradycardia in SHR was eliminated by additional pre-treatment with atropine, resulting in a minor tachycardia, which was clearly less than that observed in the control or atropine-only groups (*P* < 0.001). These observations demonstrated that the 3,4-DAP-induced parasympathetic activation was present also during the late part of the observation period in SHR. The β_1+2_AR antagonist nadolol eliminated the 3,4-DAP-induced tachycardia in both strains, but, unlike reserpine, did not reverse the response to bradycardia in SHR (Figure 3). An eliminated late HR response to 3,4-DAP was also observed after atropine + nadolol (tested in SHR only) (Figure 3).

Blocking the ganglionic nAchR with hexamethonium (Figure 1) reduced the late 3,4-DAP-induced tachycardia in WKY, whereas a reduction was not detected in SHR (Figure 2), showing that the activation of sympathetic nerve transmitter release depended in part on activation of sympathetic ganglia in WKY, but not in SHR. The augmented impact of *K*_V_ in parasympathetic ganglia in SHR was also demonstrated by the enhanced development of the 3,4-DAP-induced tachycardia after pre-treatment with hemicholinium-3, hexamethonium, or atropine, with an elevated ΔHR in the 10- to 20-min interval (*P* < 0.0001) (Figure 2). The same was observed after quinidine in SHR, although with an elevated ΔHR also after 25 min (Figure 2).

### The BP and TPR Response to 3,4-DAP

**Figure 4:**
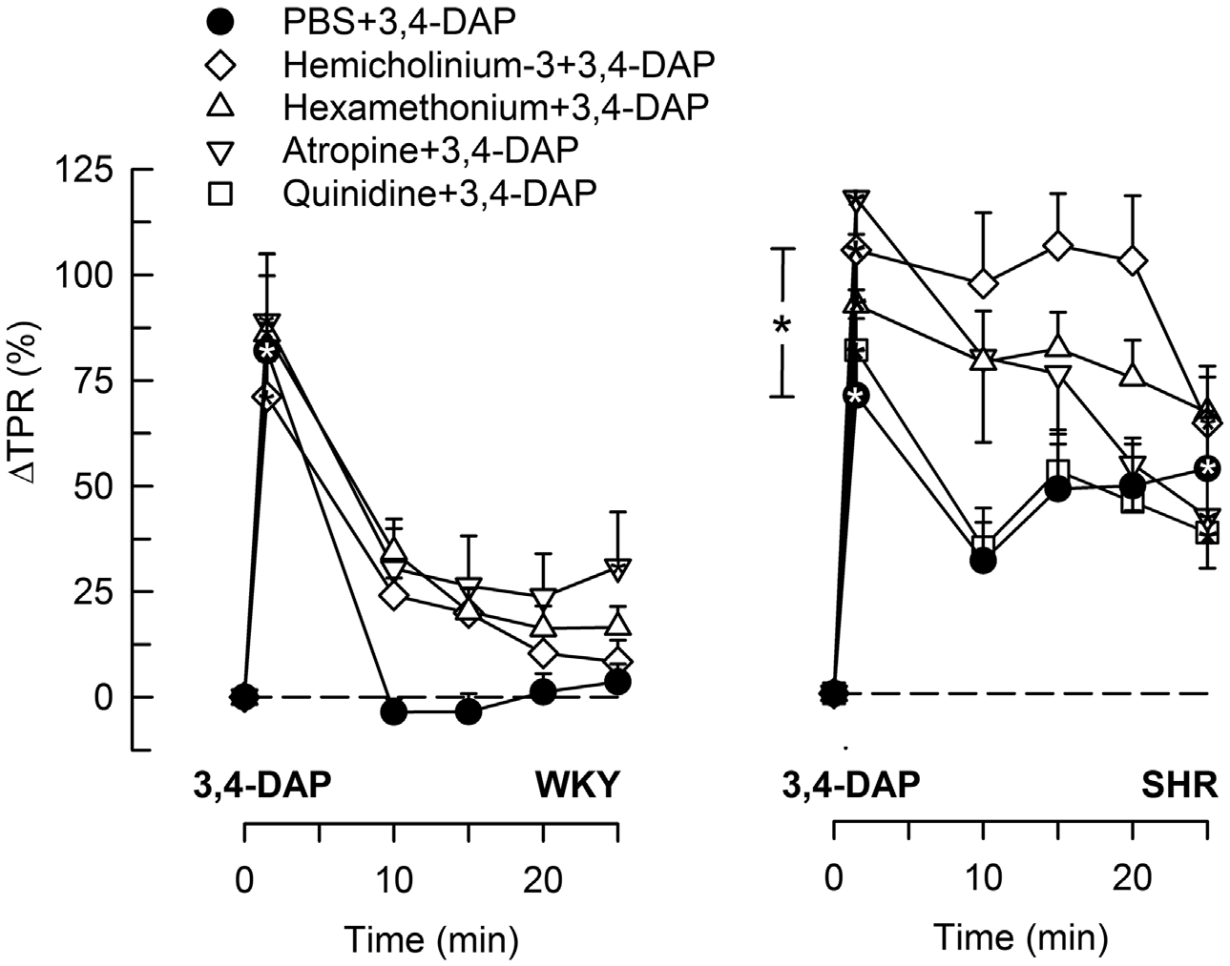
**The TPR response to 3,4-DAP after pre-treatment with cholinergic inhibitors or quinidine**. The effect of quinidine was not tested in WKY. Significant responses (* within symbol) and group differences (* in brackets) at 1 (left of curves) and 25 min (right of curves) were located as indicated. *P < 0.025 after curve analyses.

**Figure 5:**
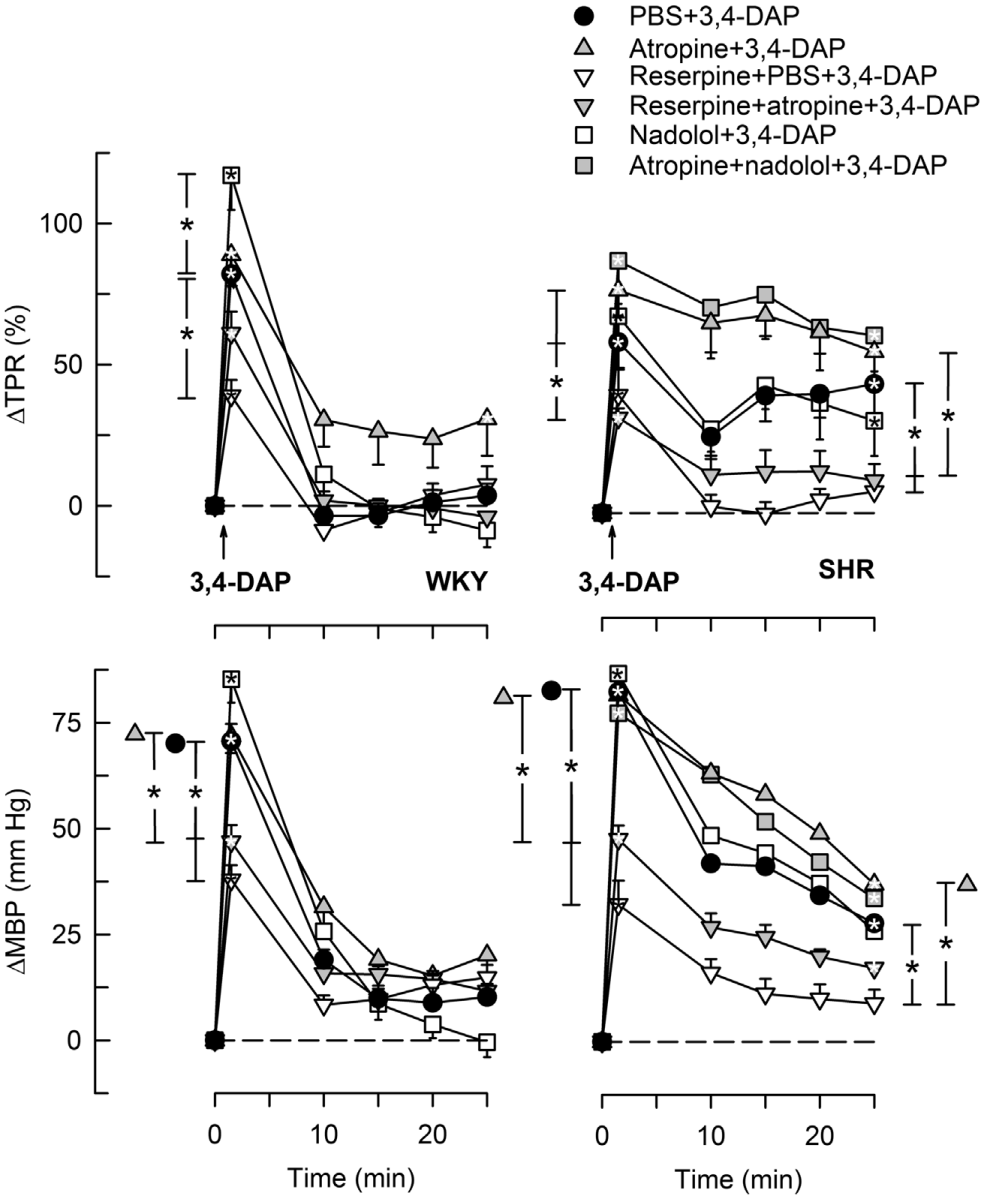
**The TPR and MBP response to 3,4-DAP after pre-treatment with reserpine or nadolol, alone or combined with atropine**. The effect of nadolol + atropine was not tested in WKY. Significant responses (* within symbol) and group differences (* in brackets) at 1 (left of curves) and 25 min (right of curves) were located as indicated. Comparisons were made between the control and the experimental groups, and between the groups pre-treated with reserpine/atropine and reserpine + atropine, and between the nadolol- and atropine + nadolol-treated groups. *P < 0.025 after curve analyses.

During the immediate response to 3,4-DAP, there was also an immediate rise in MBP and TPR in both strains (*P* < 0.0001), with no strain-related differences detected (Figures 4 and 5). Hemicholinium-3, hexamethonium, and atropine had no effect on the immediate TPR response to 3,4-DAP in WKY. These drugs increased ΔTPR in SHR, but the difference was statistically significant for hemicholinium-3 only (Figure 4). Reserpine reduced the immediate TPR response to 3,4-DAP in WKY and ΔMBP in both strains (*P* ≤ 0.005) (Figure 5), demonstrating that NE release contributed in part to the rise in TPR in WKY. ΔTPR was reduced after reserpine + atropine in SHR. The immediate TPR response was enhanced after pre-treatment with nadolol in WKY but not in SHR (Figure 5).

The vasoconstrictory TPR response to 3,4-DAP quickly returned to pre-injection levels in WKY, but remained high in SHR (*P* < 0.001). The elevated late response in SHR was eliminated by reserpine, also in the presence of atropine (*P* ≤ 0.012), but was not influenced by nadolol (Figure 5), by atropine alone, hemicholinium-3, hexamethonium, or quinidine (Figure 4). Reserpine also reduced the MBP response throughout the 3,4-DAP-observation period in SHR (Figure 5). These observations demonstrated that the elevated, late TPR response to 3,4-DAP in SHR was due to activation of neuronal NE release, independent of ganglion transmission.

### The Cardiovascular Response to 4-AP

**Figure 6:**
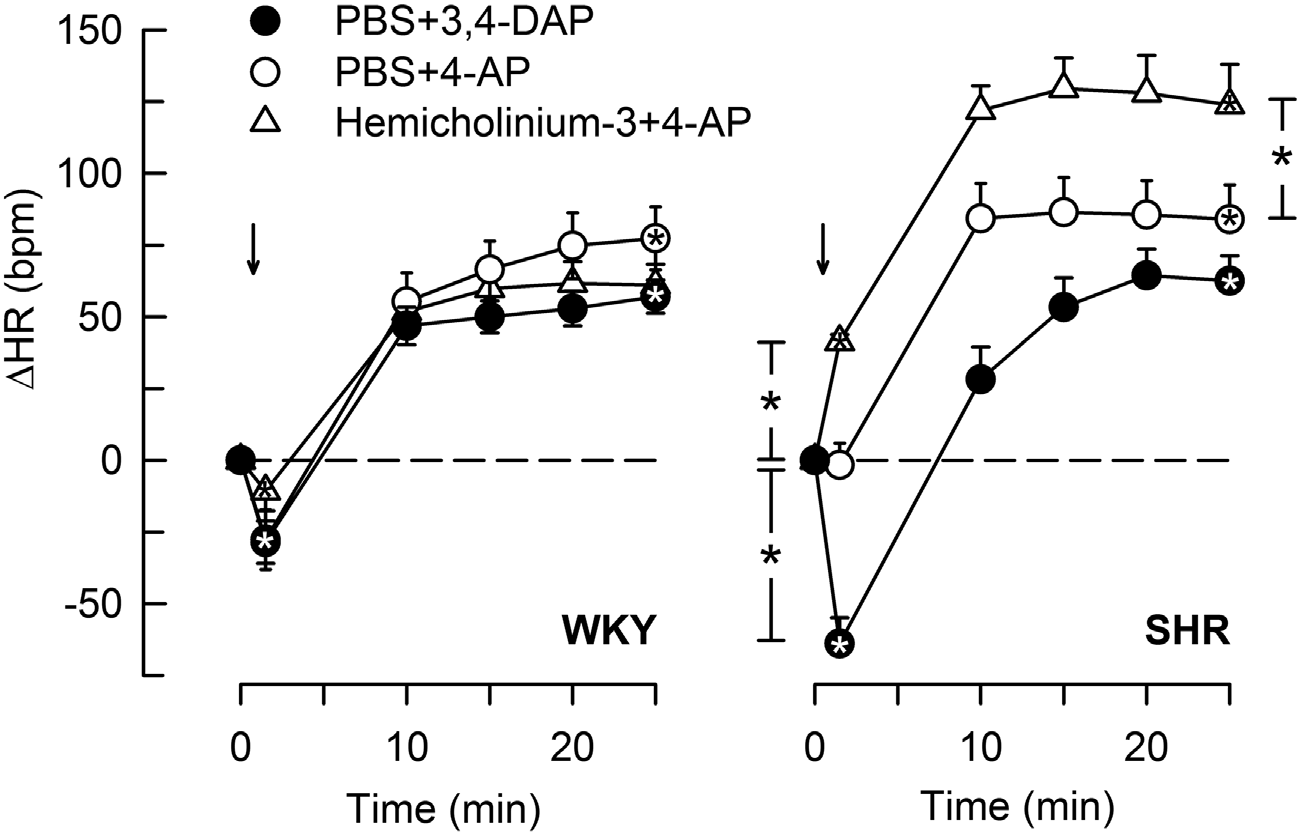
**The HR response to 4-AP compared to that following 3,4-DAP, and the effect of Ach synthesis inhibitor (hemicholinium-3) on the response to 4-AP**. Arrows indicate the injection of 4-AP or 3.4-DAP. Significant responses (* within symbol) and group differences (* in brackets) at 1 (left of curves) and 25 min (right of curves) were located as indicated. *P < 0.025 after curve analyses.

4-Aminopyridine induced a minor, initial bradycardia in WKY, not different from that following 3,4-DAP (Figure 6). There was no immediate change in HR in response to 4-AP in SHR. The tachycardia 25 min after 4-AP and 3,4-DAP did not differ in either strain. Pre-treatment with hemicholinium-3 resulted in an immediate 4-AP-induced tachycardia (*P* < 0.0001), and also an enhanced late tachycardia (*P* = 0.001) in SHR, whereas hemicholinium-3 had no significant effect on the HR response to 4-AP in WKY.

The TPR response to 4-AP did not differ from that observed in response to 3,4-DAP in either strain, and hemicholinium-3 did not significantly alter the TPR response to 4-AP (data not shown).

### The Cardiovascular Response to Nicotine

**Figure 7:**
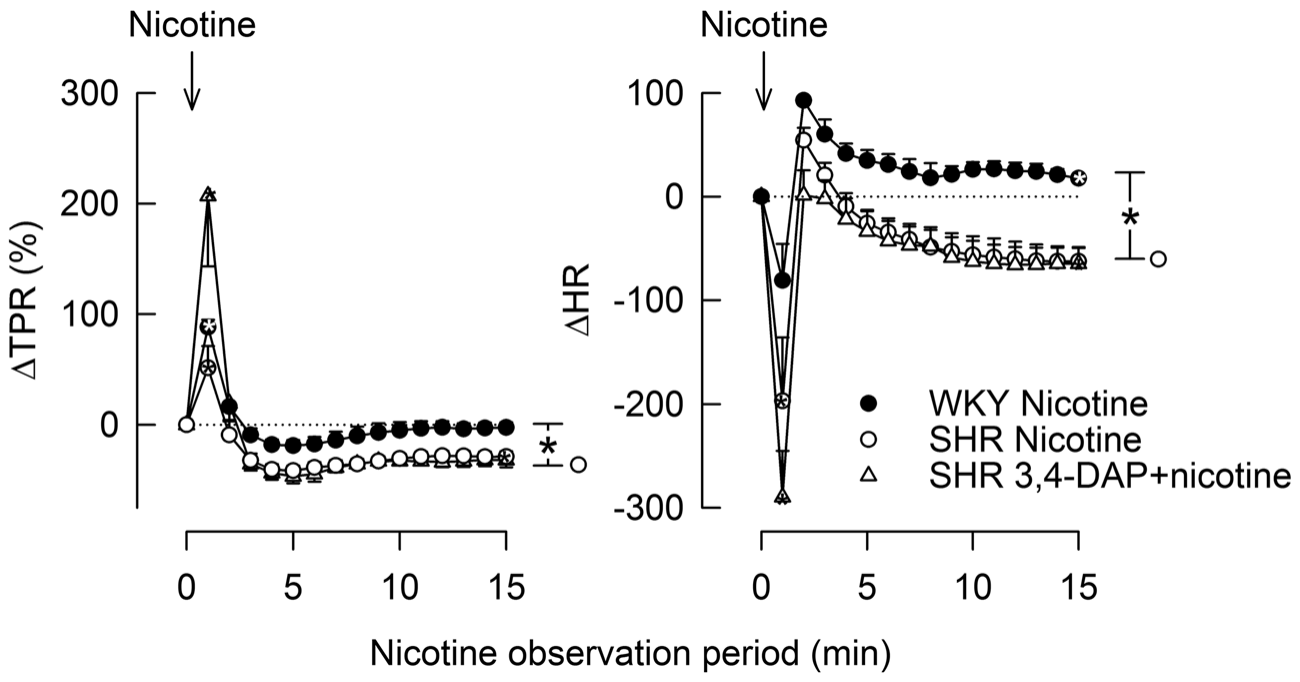
**The TPR and HR responses to nicotine, alone and after prior administration of 3,4-DAP**. Arrows indicate the injection of nicotine. The immediate TPR response was recorded at the peak response, whereas the HR response was recorded at the nadir response, occurring a few seconds earlier. *P < 0.025 after curve analyses.

Stimulation of postganglionic nAchR (Figure 1) with nicotine induced a sharp, but transient rise in MBP and TPR in both strains (Figure 7). TPR returned to pre-nicotine levels in WKY but to below baselines in SHR (*P* = 0.01). During the initial response, there was also a sharp, transient reduction in HR, which was statistically significant in SHR only (*P* = 0.024), reaching its nadir level some seconds prior to the MBP/TPR-peak response. After a rebound, a slowly developing, sustained bradycardia was observed in SHR (*P* = 0.02), whereas a tachycardia was seen in WKY (*P* = 0.006 after 15 min). Prior administration of 3,4-DAP had no effect on the TPR or HR response to nicotine (tested in SHR only).

### 3,4-DAP- and 4-AP-Induced Salivation

Salivation induced by 3,4-DAP was greater than that after 4-AP. For both drugs, salivation was lower in SHR than in WKY (*P* ≤ 0.004) (Table 2). The kallikrein concentration and total amount secreted in 3,4-DAP-induced saliva as an indication of sympathetic activation, was higher in SHR than in WKY, and in both strains the total amount secreted was greater than that in 4-AP-induced saliva (*P* ≤ 0.01). Salivation was almost totally eliminated in all groups given atropine as part of the pre-treatment (*P* < 0.001), showing that saliva volume depend on parasympathetic activation. Hexamethonium reduced salivation in WKY, indicating involvement of parasympathetic ganglion activation, but did not change the kallikrein concentration. In SHR, hemicholinium-3 and hexamethonium reduced both salivary flow and kallikrein secretion, showing involvement of parasympathetic and sympathetic ganglion transmission, respectively. Reserpine had no effect on 3,4-DAP-induced salivation in either strain, but reduced the kallikrein concentration and total amount secreted (*P* ≤ 0.05). Still, salivary flow depended also on βAR activation since nadolol reduced saliva volume to about 1/3 in WKY and almost totally abolished the salivation in SHR (*P* ≤ 0.008). Quinidine did not alter the 3,4-DAP-induced salivation (tested in SHR only, kallikrein not measured).

**Table 2:** **4-AP- and 3,4-DAP-induced salivation, salivary kallikrein concentration, and total amount kallikrein secreted**.

	WKY			SHR		
	Saliva volume (μl)	Kallikrein concentration (U/ml)	Kallikrein total secreted (U)	Saliva volume (μl)	Kallikrein concentration (U/ml)	Kallikrein total secreted (U)
PBS + 4-AP	39 ± 8	121 ± 40	4 ± 0	12 ± 4*	372 ± 87	17 ± 6
Hemicholinium-3 + 4-AP	75 ± 30	14 ± 5	1 ± 0	1 ± 1	Not measured	
PBS + 3,4-DAP	620 ± 102^†^	50 ± 15	27 ± 5^†^	230 ± 38*^†^	1096 ± 427*	380 ± 151*^†^
Hemicholinium-3 + 3,4-DAP	561 ± 35	Not measured		93 ± 21^‡^	14 ± 9^‡^	2 ± 2^‡^
Hexamethonium + 3,4-DAP	343 ± 47^‡^	34 ± 8	10 ± 1^‡^	114 ± 52	142 ± 31^‡^	36 ± 11^‡^
Atropine + 3,4-DAP	49 ± 6^‡^	91 ± 9^‡^	4 ± 2^‡^	17 ± 7^‡^	Not measured	
Reserpine + 3,4-DAP	590 ± 77	20 ± 3^‡^	12 ± 2^‡^	234 ± 4	42 ± 10^‡^	9 ± 3^‡^
Reserpine + atropine + 3,4-DAP	24 ± 10^‡^	Not measured		0 ± 0^‡^	Not measured	
Nadolol + 3,4-DAP	307 ± 83^‡^	Not measured		17 ± 10^‡^	Not measured	
Atropine + nadolol + 3,4-DAP	Not done			7 ± 4^‡^	Not measured	
Quinidine + 3,4-DAP	Not done			335 ± 79	Not measured	

Saliva was collected from the oral cavity throughout the 25-min observation period. Significant differences between the WKY and SHR 4-AP/3,4-DAP control groups (*), between corresponding 4-AP and 3,4-DAP control groups (^†^), and between corresponding control and experimental groups (^‡^), were detected as indicated.

*, ^†^, ^‡^*P* < 0.05.

### Changes in the Plasma Catecholamine Concentrations

In these experiments, re-uptake of NE was not blocked, and the changes in the plasma NE concentration were therefore small (Table 3). However, the concentration of NE at the end of the 25-min 3,4-DAP/4-AP-observation period was in both strains greater than that in time controls given PBS only (*P* ≤ 0.007) and was for both drugs higher in SHR than in WKY (*P* ≤ 0.008). The NE concentration after 4-AP was slightly higher than that after 3,4-DAP in WKY (*P* = 0.005). The plasma concentration of epinephrine in the 4-AP and 3,4-DAP controls did not differ from that in the time controls.

**Table 3:** **The concentration of catecholamines in plasma after 4-AP, 3,4-DAP, and nicotine**.

Groups	WKY		SHR	
	Norepinephrine (nM)	Epinephrine (nM)	Norepinephrine (nM)	Epinephrine (nM)
Time controls (PBS + PBS)^a^	0.5 ± 0.1	8.1 ± 1.5	1.2 ± 0.2^‡^	13.2 ± 2.5
PBS + 4-AP	2.2 ± 0.2*	10.2 ± 2.1	8.3 ± 1.0*^,‡^	9.5 ± 1.8
PBS + 3,4-DAP	1.4 ± 0.1*^†^	10.1 ± 1.8	6.0 ± 0.7*^,‡^	20.2 ± 4.7
Hemicholinium-3 + 3,4-DAP	0.8 ± 0.2^├^	4.2 ± 2.6	2.7 ± 0.4^├^	5.3 ± 1.4
Hexamethonium + 3,4-DAP	Not measured		2.5 ± 0.3^├^	3.4 ± 0.9^├^
Atropine + 3,4-DAP	Not measured		12.9 ± 3.1^├^	18.6 ± 7.8
Reserpine + 3,4-DAP	0.0 ± 0.0^├^	5.5 ± 3.7	Not measured	
Reserpine + atropine + 3,4-DAP	0.0 ± 0.0^├^	6.9 ± 1.1	Not measured	
Nadolol + 3,4-DAP	0.3 ± 0.3^├^	5.5 ± 0.8	6.8 ± 2.6	14.7 ± 8.9
Atropine + nadolol + 3,4-DAP	Not done		9.0 ± 1.8	54.0 ± 15.5
Quinidine + 3,4-DAP	Not done		4.1 ± 1.1^├^	11.4 ± 3.2
Nicotine	2.1 ± 0.3*	3.1 ± 0.7*	1.4 ± 0.3	3.7 ± 2.1*
3,4-DAP + nicotine	Not done		12.0 ± 1.3^├^	26.1 ± 6.0

Significant differences between the time-control and the 4-AP/3,4-DAP/nicotine control groups (*), between the 4-AP and 3,4-DAP control groups within each strain (^†^), between corresponding WKY and SHR PBS/4-AP/3,4-DAP controls (^‡^ after SHR values), and between corresponding 3,4-DAP control and experimental groups (^├^), were detected as indicated.

*^a^From Ref. (10), run in part intermittently with the present 4-AP and 3,4-DAP control groups. *N* = 4–9 rats per group, but 20 in the 4-AP-groups*.

*, ^†^, ^‡^, ^├^*P* < 0.05.

NE was not detected in plasma from reserpinised WKY (not measured in SHR), whereas the concentration of epinephrine was not altered. Pre-treatment with nadolol reduced the concentration of NE in WKY (*P* ≤ 0.018) but not in SHR, and had no significant effect on the concentration of epinephrine. The concentration of both catecholamines was reduced in both strains after hemicholinium-3 and hexamethonium, although the difference was not statistically significant for all. Pre-treatment with atropine (measured in SHR only) increased the concentration of NE but had no effect on the plasma epinephrine concentration. Atropine + nadolol (tested in SHR only) and nadolol did not alter the effect of 3,4-DAP on the plasma catecholamine levels.

The concentration of NE in plasma collected 15 min after the injection of nicotine was higher than that in the time controls in WKY (*P* = 0.009) but not in SHR, whereas the concentration of epinephrine was lower than in the time controls in both strains. The concentration of NE but not epinephrine was higher in SHR after 3,4-DAP + nicotine than after 3,4-DAP alone.

## Discussion

The main result in the present study was that 3,4-DAP-induced inhibition of stabilizing, hyperpolarizing *K*_V_ currents activated Ach release and nAchR signaling in cardiac parasympathetic ganglia, and thus precipitated a negative chronotropic effect that was blocked by atropine. This response was greater and of longer duration in SHR than in WKY. This activated parasympathetic component was opposed by the positive chronotropic response induced by NE release, activated by inhibition of *K*_V_ currents, first in sympathetic ganglia and subsequently in sympathetic nerve endings.

Blockade of hyperpolarizing *K*_V_ currents with 3,4-DAP precipitated transmitter release from postganglionic, parasympathetic, and sympathetic, cardiac nerves. This was demonstrated by the biphasic HR response to 3,4-DAP, i.e., an initial bradycardia followed by tachycardia, eliminated by the mAchR antagonist atropine and the sympatholytic agent reserpine, respectively (Figure 1). The parasympathetic bradycardia involved activation of parasympathetic ganglion transmission, with Ach release from preganglionic neurons and subsequent activation of nAchR in the postganglionic neuron. This was concluded since the 3,4-DAP-induced bradycardia was also eliminated by the Ach synthesis inhibitor hemicholinium-3 and the nAchR antagonist hexamethonium (Figure 1). When the influence of this parasympathetic activation was inhibited by atropine, which does not block ganglion transmission, the initial bradycardia was replaced by tachycardia. Thus, 3,4-DAP in addition activated sympathetic ganglion transmission that elicited a positive chronotropic component. This conclusion was further confirmed by that 3,4-DAP induced neither bradycardia nor tachycardia after pre-treatment with reserpine combined with atropine. Compatible with a counter-acting effect of the simultaneous parasympathetic and sympathetic activation, reserpine enhanced the initial, 3,4-DAP-induced bradycardia in SHR.

The late 3,4-DAP-induced tachycardia appeared to involve some activation of sympathetic ganglion transmission in WKY, since ΔHR at the end of the 3,4-DAP-observation period was lowered by hexamethonium. However, this was not the case in SHR, where the late reserpine-sensitive tachycardia, unlike the initial response, was not reduced by hemicholinium-3 or hexamethonium. The late tachycardia was therefore in this strain precipitated exclusively by NE release due to inhibition of *K*_V_ in the postganglionic sympathetic nerve terminals.

Since 3,4-DAP is a *K*_V_ blocker, the parasympathetic component activated by 3,4-DAP demonstrated the impact of inhibitory, hyperpolarizing *K*_V_ currents on parasympathetic ganglion Ach release (Figure 1). The role of these *K*_V_ currents was far more pronounced in SHR than in WKY. This was clearly demonstrated by the strongly increased bradycardia during both the initial and the late HR response to 3,4-DAP in SHR when the sympathetic influence had been eliminated by reserpine. In addition, removal of this parasympathetic component by hemicholinium-3, hexamethonium, or atropine enhanced the 3,4-DAP-induced tachycardia in the interval between 10 and 20 min in SHR but not in WKY. Thus, the inhibitory effect of 3,4-DAP-induced parasympathetic ganglion activation on the positive chronotropic response to NE release was sustained in SHR, different from the transient effect in WKY, demonstrating enhanced *K*_V_ inhibition of ganglion transmission in SHR. Augmented *K*_V_ currents in the preganglionic nerve terminal will stabilize the neuron and hamper parasympathetic ganglion transmission (Figure 1), thus functioning as a bottleneck to parasympathetic ganglion transmission, similar to that described for parasympathetic ganglia in myocardial infarction (8). The resulting parasympathetic hypoactivity will therefore cause HR to be controlled predominantly by the adrenergic component, compatible with that observed in SHR and hypertensive patients (1–3). The cardiac parasympathetic ganglia are located in clusters in fat tissue in the atrial epicardium and in the atrial and ventricular septa [reviewed in Ref. (28)], where they control vagal trafficking to the heart. Electrical stimulation of the sinoatrial-innervating ganglion elicited bradycardia, whereas the atrioventricular-innervating ganglion did not (29). The enhanced *K*_V_ inhibition of parasympathetic ganglion transmission in SHR may therefore reside within sinoatrial-innervating ganglion cells.

The peripherally restricted β_1+2_AR antagonist nadolol eliminated the 3,4-DAP-induced tachycardia in both strains, compatible with the fact that the tachycardia depended on 3,4-DAP-induced NE release, as demonstrated by reserpine. However, different from that after reserpine, pre-treatment with nadolol did not precipitate a sustained 3,4-DAP-induced bradycardia. This difference was most likely explained by the fact that the negative chronotropic effect of vagal stimulation is largely dependent on the presence of sympathetic activity (9). Blockade of the cardiac βAR therefore eliminated the substrate for the parasympathetic influence. This differed from that after reserpine, where circulating epinephrine apparently maintained βAR activation. However, nadolol had no effect on the initial, atropine-sensitive 3,4-DAP-induced bradycardia, which may be explained by the more slowly developing sympathetic activation, thus allowing the parasympathetic activation to dominate the HR response in this early phase. The conditions, which elicit the elevated *K*_V_ inhibition of parasympathetic ganglion transmission in SHR, may therefore preferably be studied in reserpinized rats, where its influence on HR control is easily detectable.

An effect of 3,4-DAP on Ach release from the preganglionic parasympathetic neuron, with subsequent stimulation of postsynaptic nAchR in the postganglionic neuron, was supported by the fact that 3,4-DAP did not influence the HR response to nicotine in SHR. However, the HR response to nicotine differed in the two strains, with an acute bradycardia, which, after a rebound, was followed by a sustained bradycardia in SHR, but by tachycardia in WKY. The bradycardia in SHR was likely to result from an increased number and/or sensitivity of the nAchR in order to compensate for the *K*_V_-dependent reduction in Ach release from the parasympathetic preganglionic neuron (Figure 1). A similar argument may also explain the late reduction in TPR following nicotine in SHR. The slowly developing, transient tachycardia seen in WKY may involve presynaptic nAchR, which enhanced sympathetic nerve NE release (30), in agreement with the slightly elevated NE overflow after nicotine in this strain. The elevated NE concentration in plasma from SHR given 3,4-DAP + nicotine compared to 3,4-DAP alone was likely to result from similar mechanisms or from the prolonged action of 3,4-DAP in the former group.

Presynaptic nAchR also appeared to enhance the 3,4-DAP-induced release of NE since both hemicholinium-3 and hexamethonium but not atropine reduced the plasma concentration of NE in both strains (Figure 1). NE overflow was also reduced by nadolol in WKY, in agreement with the fact that both β_1_AR and β_2_AR may facilitate NE release (31).

Ach release and activation of nAchR also control catecholamine secretion from adrenal chromaffin cells. The secretion of epinephrine was activated by surgical stress, since the plasma epinephrine concentration was almost totally absent in plasma from rats not subjected to surgery (32), and the concentration after 3,4-DAP was not different from that in the time controls. This secretion evidently involved activation of adrenal nAchR, since both hemicholinium-3 and hexamethonium but not atropine reduced the plasma concentration of epinephrine.

3,4-DAP and 4-AP are non-selective *K*_V_ blockers, and the response to 4-AP was not influenced by inhibitors of several non-*K*_V_ K^+^ channels (10). On the other hand, 3,4-DAP will influence *K*_V_ channels also in other cells, such as cardiac pacemaker cells and cardiomyocytes, where *K*_V_ contribute to action potential repolarization. However, inhibition of such currents apparently had little impact on the HR response to 3,4-DAP, since no HR response and only a minor tachycardia was observed in WKY and SHR, respectively, after pre-treatment with reserpine + atropine. The HR response to 3,4-DAP was therefore fully explained by its effect on the two branches of the autonomic nervous system.

The initial 3,4-DAP-activated bradycardia was not likely to result from baroreceptor activation due to the simultaneous rise in BP, since a similar rise in BP following selective stimulation of NE release by tyramine, precipitated tachycardia only (19), even when analyzed at high-resolution intervals within the first minute (Berg, unpublished observations). We have previously demonstrated that baroreflex control of HR was eliminated in Nembutal anesthetized rats, as large changes in BP elicited hardly any change in HR (33, 34). Furthermore, the present results demonstrated that when the late elevated TPR and BP in SHR was eliminated by reserpine, 3,4-DAP still induced bradycardia in this interval, thus excluding a role of baroreceptor activation. The absence of bradycardia during tyramine-induced increase in TPR and the augmented bradycardia in reserpinized SHR where the TPR response to 3,4-DAP was eliminated, also indicated that the 3,4-DAP-induced bradycardia did not result from failing cardiac function due to cardiac vasoconstriction.

The antiarrhythmic drug quinidine reduced BP and HR baselines in SHR. These actions may possibly result from enhanced vagal transmission due to *K*_V_ 1.5 inhibition, parallel to that demonstrated for VSMC *K*_V_ 1.5 (22, 23). The effect of quinidine on the HR response to 3,4-DAP mimicked that of atropine, compatible with the known anti-mAchR effect of this drug (24). However, unlike atropine, the 3,4-DAP-induced tachycardia remained elevated also after 25 min after pre-treatment with quinidine. If the *K*_V_ responsible for 3,4-DAP parasympathetic ganglion activation, in fact, included *K*_V_ 1.5, this observation may possibly result from an increased dose of *K*_V_ 1.5 inhibitor.

3,4-Diaminopyridine-induced salivary flow appeared to be a pure parasympathetic response, since it was almost totally abolished by atropine but was not influenced by reserpine. However, it was also reduced in WKY and abolished in SHR by nadolol, indicating that salivation depended on a permissive βAR influence. The mechanisms responsible for this βAR influence were not clear. The 3,4-DAP-induced salivation was in both strains about 20 times greater than that induced by 4-AP, indicating that 3,4-DAP was a more efficient activator of Ach release than 4-AP. Salivation in response to 3,4-DAP was greater in WKY than in SHR. The role of parasympathetic ganglion and/or neuronal Ach release was not clear cut, but seemed in both strains to involve both levels, as indicated by the effect of hemicholinium-3, hexamethonium, or atropine. These results paralleled the augmented effect of 3,4-DAP on cardiac parasympathetic ganglion transmission compared to 4-AP, although in the heart with a greater effect in SHR than in WKY. This drug-dependent difference in the cardiac response was clearly demonstrated in reserpinised SHR, where 3,4-DAP induced a prominent bradycardia (present study) whereas 4-AP did not (10). Also, the secretion of kallikrein, which is primarily activated by α_1_AR stimulation of submandibular gland granular tubules (25), was greater after 3,4-DAP than 4-AP, and much greater in SHR than in WKY. The secretion of kallikrein in SHR involved activation of sympathetic ganglion transmission, since it was reduced after hemicholinium-3, hexamethonium, and reserpine. It therefore seemed that 3,4-DAP-activated sympathetic as well as parasympathetic ganglion transmission in the heart as well as in other organs as demonstrated here for the salivary glands, and with a variable strain-related difference for different functions.

4-aminopyridine, unlike 3,4-DAP, readily enters CNS where it stimulates sympathetic output (11–13, 15). The difference in transport across the blood–brain barrier was clearly evident by the muscular twitches due to CNS cortical stimulation, observed in response to 4-AP but not 3,4-DAP (15). However, the 4-AP-activated sympathetic control of HR did not differ from that induced by 3,4-DAP, since a drug-related difference was no longer detected when the augmented parasympathetic influence in SHR had subsided, i.e., after 25 min. This observation paralleled the similar plasma NE overflow following 4-AP and 3,4-DAP.

3,4-Diaminopyridine, like previously demonstrated for 4-AP (10, 14, 15), induced an immediate rise in TPR, transient in WKY, but sustained in SHR. The TPR-peak response to 3,4-DAP was in part reduced by reserpine in WKY and by reserpine + atropine in SHR. The vasoconstriction was therefore likely to result from inhibition of hyperpolarizing, relaxing VSMC *K*_V_ as well as from the 3,4-DAP-induced NE release, particularly in WKY. The K^+^ channels mainly found in arterial VSMC are *K*_V_1.2 and 1.5 and the high conductance, Ca^2+^-gated K^+^ channel (BK_Ca_), with BK_Ca_ playing a greater role than *K*_V_ in hypertension [for review, see in Ref. (35)]. A clear strain-related difference was not detected in the initial TPR response to 3,4-DAP (present study) or to 4-AP (10). However, the TPR response to 4-AP was increased after inhibition of protein kinase C (PKC) or inhibition of the PKC-generating α_1_AR, and angiotensin AT1 and endothelin A receptors (14), in accordance with an inhibitory effect of PKC on VSMC *K*_V_ (36). Excessive activation of PKC in SHR may therefore shift the balance from *K*_V_ to BK_Ca_ control of vascular tension. Interestingly, exercise increased *K*_V_1.2 and *K*_V_1.5- and decreased BK_Ca_-expression in SHR, paralleled by a reduction in BP (37). A shift from *K*_V_ to a BK_Ca_ control of vascular tension in SHR may explain why quinidine did not increase TPR baseline or the TPR response to 3,4-DAP in SHR (not tested in WKY). The reason for the increased immediate ΔTPR after hemicholinium-3 in SHR, with a similar tendency also after atropine, may possibly suggest an increase in *K*_V_ currents after parasympathetic inhibition in this strain. The elevated TPR response during the late part of the response to 3,4-DAP in SHR was, as that in response to 4-AP (10), eliminated by reserpine, showing that it resulted from the release of NE.

The SHR is also an animal model for attention deficit hyperactivity disorder (ADHD). However, the present results were performed on anesthetized rats. In addition, ADHD results from changes in the CNS, whereas 3,4-DAP does not cross the blood–brain barrier and has a peripheral action exclusively. It therefore did not seem likely that this comorbidity directly influenced the present results.

## Conclusion

The present study showed that blocking *K*_V_ with 3,4-DAP induced Ach release in cardiac parasympathetic ganglia with a far greater and more sustained effect on HR in SHR than in WKY. It could therefore be deduced that parasympathetic ganglion transmission was inhibited in this strain by an augmented hyperpolarizing *K*_V_ current, which stabilized the neurons and prevented presynaptic Ach release and activation of the postsynaptic ganglion nAchR. This enhanced *K*_V_ activity was therefore likely to preclude the protective effect of vagal function, as it has been observed in hypertension and also congestive heart failure (38, 39). A resulting cardiac sympathetic hyperactivity may lead to hypertensive left ventricular hypertrophy (40), an independent predictor of morbidity and mortality (41). The mechanism underlying the augmented parasympathetic ganglion *K*_V_ currents and, thus, vagal dysfunction in SHR is as yet not known.

## Conflict of Interest Statement

The author declares that the research was conducted in the absence of any commercial or financial relationships that could be construed as a potential conflict of interest.
